# Genetic insights into non-syndromic Tetralogy of Fallot

**DOI:** 10.3389/fphys.2022.1012665

**Published:** 2022-10-06

**Authors:** Nouf J. Althali, Kathryn E. Hentges

**Affiliations:** ^1^ Division of Evolution, Infection and Genomics, School of Biological Sciences, Faculty of Biology, Medicine and Health, Manchester Academic Health Sciences Centre, University of Manchester, Manchester, United Kingdom; ^2^ Biology Department, Science College, King Khalid University, Abha, Saudi Arabia

**Keywords:** Tetralogy of Fallot, non-syndromic, genetics, congenital heart disease, NOTCH1, FLT4, transcription factors

## Abstract

Congenital heart defects (CHD) include structural abnormalities of the heart or/and great vessels that are present at birth. CHD affects around 1% of all newborns worldwide. Tetralogy of Fallot (TOF) is the most prevalent cyanotic congenital cardiac abnormality, affecting three out of every 10,000 live infants with a prevalence rate of 5–10% of all congenital cardiac defects. The four hallmark characteristics of TOF are: right ventricular hypertrophy, pulmonary stenosis, ventricular septal defect, and overriding aorta. Approximately 20% of cases of TOF are associated with a known disease or chromosomal abnormality, with the remaining 80% of TOF cases being non-syndromic, with no known aetiology. Relatively few TOF patients have been studied, and little is known about critical causative genes for non-syndromic TOF. However, rare genetic variants have been identified as significant risk factors for CHD, and are likely to cause some cases of TOF. Therefore, this review aims to provide an update on well-characterized genes and the most recent variants identified for non-syndromic TOF.

## Introduction

**FIGURE 1:**
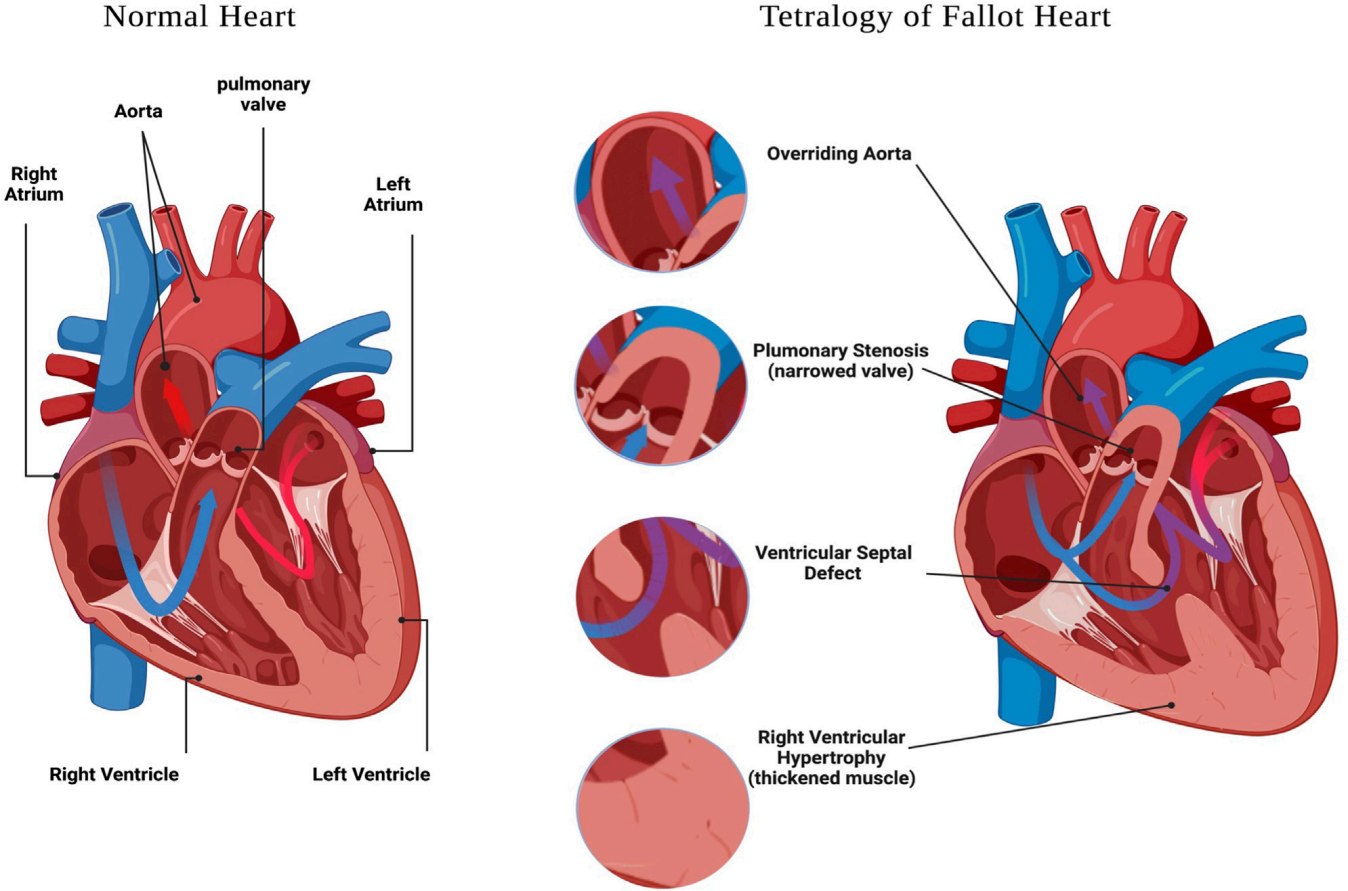
Tetralogy of Fallot features. A schematic representation of a heart with: Ventricular Septal Defect, Overriding Aorta, Pulmonary Stenosis and Right Ventricular Hypertrophy. Created with BioRender.com.

Congenital heart defects (CHD) are structural malformations of the heart or/and great vessels which affect about 1% of live born infants (Hoffman and Kaplan, 2002). Globally, CHDs are the greatest cause of death from non-communicable diseases in individuals under 30 years of age, and are also the leading cause of mortality from congenital abnormalities (Su et al., 2022). In a study conducted between 2000 and 2005 in Europe, it was reported that approximately 88% of CHD cases are not associated with chromosomal alterations (Dolk et al., 2011), suggesting other genetic defects are responsible for the majority of CHD in this population. Within CHD, Tetralogy of Fallot (TOF) is the most common cyanotic congenital heart defect, affecting 3 per 10,000 live births, and comprising 5–10% of all congenital heart abnormalities (Suzuki et al., 1996; Bailliard and Anderson, 2009; Postoev et al., 2014); and (Park and Thayer, 2014). The hallmark characteristics of TOF include four structural alterations to the heart and great vessels (Figure 1), which are: pulmonary stenosis, ventricular septal defect (VSD), overriding aorta, and right ventricular (RV) hypertrophy (Shinebourne et al., 2006; Bailliard and Anderson, 2009); and reviewed in (Bedair and Iriart, 2019).

### Pathophysiology of TOF

The TOF heart anatomy permits blood to be mixed between the pulmonary and systemic circulations. This mixture commonly happens at the VSD, where a right-to-left shunt introduces deoxygenated blood into the systemic circulation, resulting in cyanosis. The differential pressure gradient between the RV and LV determines the right-to-left shunt across the VSD. The degree of the right ventricular outflow tract obstrucrtion (or pulmonary stenosis) determines the quantity of pulmonary blood flow (the RV stroke volume) (Wilson et al., 2019). Understanding the dynamical variables that aggravate or improve the right-to-left shunt is critical to understanding the essential care of disease or preoperative neonates with TOF (Wilson et al., 2019).

An irregular blood flow pattern, chamber hypoplasia, obstructive lesions and aberrant venous and arterial connections found in individuals with CHD results in some of the most interesting and complicated abnormalities in normal heart physiology (Škorić-Milosavljević et al., 2021). The physiological changes surrounding hyper cyanotic incidents consist of either a reduction in systemic vascular resistance or a rise in pulmonary resistance, both of which paticipate to a right-to-left shunt across the VDS (Mcleod et al., 2018; Ho et al., 2019); and (Senst et al., 2022). Due to the right ventricle to left ventricle flow shunt and high flow levels through the overriding aorta, the endocardium encompassing the VSD faces higher wall shear stresses. In addition, the TOF right ventricle and left ventricle have higher pressures than normal hearts, but only the TOF right ventricle has higher wall shear stresses and thickening (Wiputra et al., 2018). Understanding the blood flow dynamics in TOF hearts, most likely due to anatomical differences and their different illnesses, could aid in future minimally invasive foetal heart therapies.

Surgical repair of the morphological and physiological alterations in the TOF heart has markedly increased survival rates for infants with TOF. Repair in the first year of life is preferable, and earlier repair improves survival outcomes (Peck et al., 2021). Although patients with repaired TOF have a high chance of surviving into adulthood, the transition into middle life is accompanied by severe morbidity and increased mortality rates (Dennis et al., 2017). Both atrial and ventricular arrhythmias are a common source of morbidity and mortality following TOF repair (Pinsker et al., 2022). Additional causes of mortality for individuals with TOF are heart failure and sudden cardiac death (Norgaard et al., 1996; Oechslin et al., 2000); and (Dennis et al., 2017). Sudden mortality in the repaired TOF population is widely documented, with multiple risk factors, which are assumed to be related to chronic RV volume overload over time.

### Genetics of TOF

A higher incidence of CHD in first-degree relatives and children of TOF patients (Postoev et al., 2014) suggests a genetic aetiology to this disorder. The genetic analysis of syndromic TOF patients has offered valuable insights into causal genes in specific individuals. Approximately 20% of cases are linked to a recognised condition or chromosomal abnormality (Bailliard and Anderson, 2009). These TOF cases are found in patients with genetic syndromes including 22q11 microdeletions, trisomy 21, Alagille’s disorder, Cat Eye syndrome, or CHARGE and VATER/VACTERL syndromes (Goldmuntz et al., 1998; Goldmuntz, 2001; McElhinney et al., 2002; Michielon et al., 2006); and (Marino et al., 2012). About 80% of TOF cases are non-syndromic, and display no indication of familial Mendelian CHD segregation, with no identified aetiology (Goodship et al., 2012; Palomino Doza et al., 2013). Rare, *de novo* genetic variations have been found as substantial factors in TOF (Jin et al., 2017). Notably, variants in the key cardiac transcription factor *NKX2.5* were the first to be identified as a genetic cause of TOF (Goldmuntz et al., 1998; Benson et al., 1999). Subsequent studies have further extended our knowledge of genetic alterations resulting in TOF. Therefeore, this mini review aims to offer an update on the genes and variants reported to be associated with non-syndromic TOF (Table 1). As highlighted in this review, disruption of the balanced interaction of transcription factors which are crucial for cardiogenesis plays a substantial role in the aetiology of non-syndromic TOF.

**TABLE 1:** Non-syndromic TOF genetic variants.

Name of the gene	Variant or genetic alteration	Cardiac phenotype	Role to TOF	References
*NOTCH1*	p.R448X, p.W1638X and p.Q1733X	TOF	These variants cause loss of protein function. Some are premature stop codons, single base pair deletions causing frameshifts and premature termination, or a single nucleotide deletion in a consensus splice site sequence	Page et al. (2019)
	p.G115fsX6, p.N147fsX128 p.C1322fsX121 (c.5385-1delC)			
	p.G200V p.C292Y p.Q1495K, p.C1549Y p.N1875S and several other variants			
	p.C283G, p.Q415K, p.E606K, p.N623K, p.C682Y, p.D710N, p.C815Y, p.C1536R	TOF	Eight ultra-rare missense *NOTCH1*variants would support aberrant vascular development and related signalling as probable causes in TOF.	Manshaei et al. (2020)
*FLT4*	p.30RfsX3, p.R82X, T168RfsX18, Y361X, P363PfsX64, Q736X, L935PfsX82, P948PfsX54, Q999X	TOF and/or PS, APVS, RAA, PA	Loss of *FLT4* function in TOF with evidence of incomplete penetrance	Jin et al. (2017)
	p.Y361X, p.Y369X, p.E896X, p.Q920X, p.R1031X, p.Q1126X p.P363fsX25, p.Q423fsX3, p.L636fsX3, p.Y853fsX20, p.N905fsX20 and p.Y1337fsX19 (c.3002–1C>T, c. 3002-2T>C, c.2300C>G and c.2849del21		These variants were loss of function, which include premature stop codons,frameshifts and premature truncation and splice variants	Page et al. (2019)
*NKX2.5*	c.73C>T, p.R25C, c.63A>G, pE21E	TOF/ASD	c.73C>T is considered the most recurrent *NKX2.5* variant in CHD patients (Kalayinia et al., 2021). The results suggested a potential impact of these variants on splicing	EL Bouchikhi et al. (2021)
	Several variants were reviewed in (Chung and Rajakumar, 2016)	TOF/ASD/Atresia/Stenosis	The most widely accepted hypothesis is that all molecules involved in cardiac development are reduced. However, additional research is needed to determine how the novel mutations identified in *NKX2.5* affect transcription factor function and thus heart development	Chung and Rajakumar (2016)
*GATA4*	c.1220C>A, p.P407Q; c.1138G>A, p.V380 M	TOF and patent foramen ovale (PFO)	Variant p.V380M was found in *GATA4*’s second transcription activation TAD domain. p.P407Q was identified as pathogenic in the ClinVar and Human Genome Mutation Database (HGMD)	Qian et al. (2017)
*GATA5*	c.830C>T; p.P277L	TOF; overriding aorta, loss of the ventricular septum and aortic anterior wall, indicating the presence of a VSD in the foetus	This variant is pathogenic due to inhibiting *GATA5* nuclear translocation	Jin et al. (2022)
	c. 943T>A, p.S315T, c.274G>T, p.A92S	TOF and/or PFO	These novel variants were found in the N- and C-termini of *GATA5*, as well as the functional domains, and may increase the risk of cardiac abnormalities	Qian et al. (2017)
	there are at least 31 identified GATA5 variations in TOF patients, containing 29 missense variants and two non - protein coding variants	TOF and/or VSD, BAV, aortic stenosis	Reduce *GATA5* transcription factor activity leading to valve morphogenesis defects and BAV.	Wei et al. (2013), Qian et al. (2017), Shi et al. (2014), Kassab et al. (2015)
*GATA6*	c.331G>A, p.D111N, c.972C>G, p.H324Q	TOF	Those two novel variants were observed in the TAD domains and might raise cardiac malformations	Qian et al. (2017)
*TBX1*	p.P43_G61del PPPPRYDPCAAAAPGAPGP	TOF with patent foramen ovale (PFO) and right aortic arch, a dysplastic pulmonary valve, severe valvar and supravalvar pulmonary stenosis and confluent branch pulmonary arteries	These *TBX* family variants and *CITED2* variant, reported in this study, were novel which likely alters the protein function and may increase cardic defects but these possibilities need to be confirmed	Qian et al. (2017)
*TBX2*	c.2139dupG	TOF and AVSD		
*TBX5*	c.409G>T, p.V137L	TOF and PDA and PFO		
*CITED2*	c.-1AT	TOF and PAA		
*ZFPM2/FOG2*	c. C452T, p.T151I, c.C1208G, p.A403G, c.G1552A, p.A518T, c.A2107C, p.M703L, c.G2482A, p.V828M c.C3239T, p.S1080F	TOF/DORV	These variants might have an essential role in pathogenesis of TOF and DORV, suggesting that *ZFPM2/FOG2* genetic variations might be potential biomarkers and therapeutic targets for non-syndromic TOF and DORV.	Huang et al. (2014)
	c.3442G>A, p.E1148K; c.3014A>G, p.E1005G	TOF and/or PFO	These variants may disrupt interactions with *GATA4* causing inhibition of *GATA4* protein transcription	Qian et al. (2017)
	p.E30G, p.I227V, p.M544I, p.V339I, p.K737E, p.A611T and other variants	TOF and Double outlet right ventricle (DORV) or ventricular septal defect type of DORV	*ZFPM2* variants can cause complicated CHD, and may or may not have a serious effect on protein function	De Luca et al. (2010), Tan et al. (2011)
*FOXC1*	p.378GGGdel, p. 488AAAAdel	TOF with bicuspid pulmonary valve, valvar and subvalvar stenosis, confluent branch pulmonary arteries	These rare gene variants in the second heart field transcriptional network impact transcription activity, playing a key role in the growth of right-sided cardiovascular structures and the outflow tract	Töpf et al. (2014)
	p.P297S	TOF with moderate to severe subvalvar pulmonary stenosis, confluent branch pulmonary arteries, right aortic arch, and PFO.		
*FOXC2*	p.Q444R	TOF with moderate valvar pulmonary stenosis, mild subvalvar pulmonary stenosis, confluent branch pulmonary arteries, and pyloric stenosis		
*FOXH1*	p.S113T	TOF with severe valvar and subvalvar pulmonary stenosis, right aortic arch, and confluent branch pulmonary arteries		
*HAND2*	p.A25_A26insAA	TOF with pulmonary atresia and major aortopulmonary collateral arteries, right aortic arch, small atrial septal defect, small central pulmonary arteries, and absent thyroid gland		
*JAG1*	c.414_415dupGT c.2429C>T, p.P810L c.3312C>G, p.H1104Q	TOF with branch PS and PA. TOF with PS, PE.TOF	These variants are predicted to form premature termination codons affecting allele transcripts and resulting in 50% normal *JAG1* expression	Bauer et al. (2010)
	c.1511A>G, p.N504S, c.3038A>T, p.H1013L, c.2906T>C, p.M969T,c.806C>G, p.P269R	TOF and/or patent foramen ovale (PFO), or ASD.	This finding assumed that *JAG1* variations inhibited the transcriptional activation of HEY proteins *via* an aberrant signalling pathway and subsequently altered the transcriptional control of *GATA* family members, which may have an additional impact for the variants in these members	Qian et al. (2017)
	c.765C>T	TOF	This variant might affect gene functions; however, more investigation needs to be confirmed	Safari-Arababadi et al. (2018)
*FLNA*	c.3415C>T; p.L1139F	TOF	This variant was found in two brothers and further studies need to be performed to confirm the variant frequency in TOF patients in different populations	Kalayinia et al. (2021)
*KDR encoding VEGF Receptor 2*	p.G345W p.G537R	Absent pulmonary valve syndrome and a double outlet right ventricle-severely hypoplastic pulmonary valve, a right ventricle with two outlets, and a right-sided aortic arch	The findings suggest a role for rare *KDR* variants in TOF etiology *via* a loss-of-function mechanism	Škorić-Milosavljević et al. (2021)
*NDRG4*	p.T256M (NM_020465: c.767C>T, rs144494221)	PA, VSD, patent foramen ovale, patent foramen ovale	*NDRG4* p.T256M variant may be implicated in the aetiology of PA/VSD and TOF by affecting the regulation of cardiomyocyte proliferation	Peng et al. (2021)
		TOF, patent foramen ovale		
		TOF, patent foramen ovale, left superior vena cava		
*SMARCC2*	(NM_003075.5: c.3561del, p.L1188fs)	An enlarged aortic valve ventricular septal defect a narrowing of the right ventricle outflow tract pulmonary stenosis	It was still unclear if the patient’s congenital cardiac condition was isolated or syndromic	Sun et al. (2022)

Whole Exome Sequencing (WES) has been used effectively to find new candidate genes for CHD (Zaidi et al., 2013; Al Turki et al., 2014; Homsy et al., 2015); and (Sifrim et al., 2016). Previous research revealed that ultra-rare non-synonymous variants play a major role in the genetic aetiology of CHD, particularly TOF (Jin et al., 2017; Page et al., 2019); and (Reuter et al., 2019). Although familial cases of TOF are rare, likely due to the severity of the disease, inherited variants in the genes *GATA4*, *NKX2.5*, *JAG1*, *FOXC2*, *TBX5* and *TBX1* have been reported (reviewed in (Morgenthau and Frishman, 2018). A recent study of the largest group of non-syndromic TOF patients observed to date, using WES of 829 TOF patients, has found that the *NOTCH1* locus, followed by *FLT4*, is the most common site of genetic variants leading to non-syndromic TOF. Variants in these genes are detected in about 7% of TOF cases (Page et al., 2019). Moreover, rare variants in some specific transcription factors, such as *FOXC1* and *HAND2*, have been identified in non-syndromic TOF patients (Töpf et al., 2014). Because there are links between normal heart formation and multiple transcription factors, variants in transcription factors required during cardiac development can result in aberrant cardiac morphology or function, thus resulting in CHD.

### NOTCH family

NOTCH family proteins 1-4 are evolutionarily conserved transmembrane proteins that have a crucial impact on cell fate decisions (Andersson et al., 2011). The sequential expression of Notch components and related genes are required during vascular and cardiac development (Luxán et al., 2016). The *NOTCH* pathway is necessary for the developing heart for the regulation of vital pathways involving the formation of cardiac trabeculae and sequential activation of many *NOTCH* genes promotes myocardial development, formation, and compaction (Luxán et al., 2016).

Since *NOTCH* signalling is so significant for cardiac and vascular development, variants in genes encoding *NOTCH* signalling components cause a variety of cardiovascular abnormalities (Eldadah et al., 2001; McElhinney et al., 2002; Tomita-Mitchell et al., 2007; Garg et al., 2005; Stittrich et al., 2014; Kerstjens-Frederikse et al., 2016); and (Luxán et al., 2016). A recent study conducted by (Page et al., 2019) found five new *de novo NOTCH1* variants in the TOF cohort; three of these were missense variations, whereas the remaining two were truncating variants (Table 1): p. G200V, p. R448X, p. C1549Y, p. W1638X and p. N1875S. The differences in *NOTCH1* signalling observed were not due to the variants’ reduced mRNA expression. As a result, two variants, the p. C607Y and p. N1875S, recognised in TOF patients who underwent functional testing, were found to affect canonical *NOTCH1* signalling (Page et al., 2019). Furthermore, eight ultra-rare missense *NOTCH1* variants were found in the investigation of a 175-member TOF cohort in a study performed by (Manshaei et al., 2020). Their analysis confirmed that those variants were ultra-rare deleterious and involved in the pathogenesis of TOF, suggesting abnormal vascular development and related signalling defects as probable causes of TOF.

### FLT4

Vascular endothelial growth factor receptors (VEGFR1-3) are crucial in the development and long-term maintenance of the cardiovascular and lymphovascular systems. VEGFR3 (Vascular Endothelial Growth Factor 3), encoded by the *FLT4* gene, plays an essential and well-studied role in the development and formation of the circulatory vessels (Olsson et al., 2006; Karaman et al., 2022). Statistical analysis revealed that *FLT4* was among the most common genes predisposing individuals to non-syndromic TOF (Matos-Nieves et al., 2019). Likewise, exome sequence analysis revealed that *FLT4* and *NOTCH1* genes were the most significant genes that caused non-syndromic TOF in a cohort of 829 TOF patients (Page et al., 2019). Recent analysis of exome sequencing data from 811 TOF probands has found 14 *FLT4* loss of function variants (Reuter et al., 2019). Additionally, (Page et al., 2019); found that several *FLT4* variants in TOF patients are unique and severe, due to causing premature protein truncation and frameshifts located in the FLT4 immunoglobulin (Ig) and VEGFR3 protein kinase domains. Taken together, these findings highlight the role of *FLT4* in TOF and CHD; however, the precise mechanism of how *FLT4* variants provoke the cardiac structural alterations of TOF remains to be elucidated.

### NKX2.5

The human transcription factor *NKX2.5* is the first gene associated with human cardiac development. It sits at the 5q34-q35 chromosome five region and is abundantly expressed in the human foetal heart (Shinebourne et al., 2006). An essential role of *NKX2.5* during secondary heart field development is its interactions with the broader network of transcriptional factors that regulate cardiac differentiation and fate (Clark et al., 2013). Moreover, deletion of *Nkx2.5* has been linked to abnormalities in the structure of the embryonic heart, growth delay, and embryo mortality in mice (Lyons et al., 1995). Several studies have also reported that variants in *NKX2.5* cause a wide range of CHDs, including ASD, VSD, TOF, HLH, CoA, TGA, Double Outlet Right Ventricle (DORV), IAA, and cardiac outflow tract (OFT) anomalies; reviewed in (Chung and Rajakumar, 2016). Recently, new *NKX2.5* variants were discovered in a Moroccan cohort with non-syndromic TOF; a missense variant R25C and a synonymous variant E21E (EL Bouchikhi et al., 2021). The R25C variant is expected to impair *NKX2.5* dimerisation and impede activation of downstream target genes (Kasahara et al., 2000; Dentice et al., 2006). Bioinformatic predictions indicate that the E21E variant might impair *NKX2.5* transcript splicing by removing a whole exon from the transcript, resulting in translation of a shortened protein that is most likely inactive. Further *in vitro* functional tests are needed to validate this finding (EL Bouchikhi et al., 2021).

### Interaction of transcription factors

In mammals, the master cardiac transcription factors (TFs) such as *GATA4, TBX5, HAND2*, *KNX2.5*, and *MESP1* control the intricate and complicated process of heart development (Olsson et al., 2006). Pathological variants in essential cardiac transcription factor genes such as *GATA4, GATA5* and *GATA6* are associated with CHD (Kodo et al., 2009); Jiang et al., 2013; and (Granados-Riveron et al., 2011). Moreover (Töpf et al., 2014), reported that rare variants in the transcription factors of the second heart field, such as *HAND2, FOXC1, FOXC2, FOXH1,* and *TBX1*, can be found in non-syndromic TOF patients. These variants affect transcription activity; thereby disrupting the vital role these proteins play in the development of the outflow tract and right-sided cardiovascular components.

*Via* the general process of combinatorial interactions, a significant subset of cardiac transcription factors can regulate the intricate spatiotemporal control of heart morphogenesis and intermolecular interactions (Durocher and Nemer, 1998; Bruneau, 2008); and (Pradhan et al., 2017). For instance, physical interaction between GATA4, NKX2.5, and TBX5 activates downstream targets in a synergistic manner (Bruneau et al., 2001; Hiroi et al., 2001). Thus, mutations in these transcription factors may damage their interactions, thereby affecting protein function. Interestingly, according to a semi-quantitative study in the TOF cohort, (Qian et al., 2017); found that the interaction between the ZFPM2/FOG2 E1148K mutant protein with GATA4 was significantly damaged and was reduced by 50 per cent compared to the wild-type ZFPM2 protein (Qian et al., 2017). On the other hand, this result is not consistent with the previous findings whereby (Zhang et al., 2014) demonstrated that although the p.M703L and p. T843M ZFPM2 missense variants were assumed to be damaging, they had only a minor effect, if any, on the protein interaction with GATA4. Therefore, the impacts of individual variants may disrupt protein interactions to differing extents, or different variants may involve other unknown mechanisms to provoke CHD.

Moreover, multiple new potential candidate genes for causing TOF, that are linked to the function of FLT4, were identified, such as *CLDN9*, *CCDC168*, *SN2*, *L1TD*, *TACC3*, *CCDC36* and *TTC5* (Manshaei et al., 2020). More recently, it has been noted that twenty-three confirmed TOF genes form an interaction network with KDR/VEGFR2 and NOTCH1, acting as key nodes in the protein interaction network, each directly linking to 11 additional proteins *via* functional biological connections (Reuter et al., 2019). Reanalysis of the accuracy of these protein interactions may help to understand the interaction mechanisms and might inform therapeutic targets of TOF patients.

### New variants yet to be confirmed

*De novo* variants have a major role in the development of early-onset genetic diseases such as CHD. A screen of a CHD cohort was performed by (Bauer et al., 2010); and found that *JAG1* variants, producing functionally relevant sequence changes, were detected in 3% (2/94) of TOF cases. These individuals did not show signs of any other recognised syndromes. A recent study has concluded that a *JAG1* variant: c.765C>T, is strongly linked to TOF in Iranian patients with a ratio of 60.2% *versus* 5.7% in controls (Safari-Arababadi et al., 2018); this variant may affect gene function. However, additional investigations need to be performed to understand the mechanisms of *JAG1* alterations in TOF.

Furthermore, despite the limited sample size in a study conducted by (Manshaei et al., 2020); burden test findings indicated ultra-rare truncating variants in novel candidate genes, such as *ZFAND5* and *WNT5A*, that might be involved in the genesis of TOF. A novel *FLNA* variant, p. L1139F, was discovered in two Iranian brothers diagnosed with X-linked recessive TOF. This variant was heterozygous in their mother, whilst their father had the wild type reference allele. Hemizygosity for this variant is therefore predicted to have a role in CHD, particularly TOF (Kalayinia et al., 2021). Nevertheless, further research is required to demonstrate the role of this variant or other *FLNA* variants in TOF individuals and their frequency in different populations. These new gene associations reveal the need for further investigation of the role of the contribution of rare variants to TOF in a larger patient cohort, which might give new insights into additional genetic causes of non-syndromic TOF.

In addition, it has also been identified that the loss of function of three emerging candidate genes, *KDR, IQGAP1*, and *GDF1* may cause CHD and TOF (Reuter et al., 2019), but further clinical confirmation is needed. Highlighting the genetic variability of TOF; 16 additional patients have been identified to have one pathogenic/likely pathogenic variant (11 loss of function and five missense) in 16 CHD genes (*ARHGAP31, ATRX, CACNA1C, CHD7, CSNK2A1, DLL4, EP300, GATAD2B, KAT6A, LZTR1, NF1, NODAL, PIK3CA, RAF1, RASA1, and SMAD2*) and one patient has been identified with loss of function variants in two genes (*ASXL1* and *PSMD12*) (Reuter et al., 2019). In addition, recent studies have shown that new variants found in TOF cases in the *NDRG4* gene cause multiple heart abnormalities (Peng et al., 2021). Evidence suggests that *SMARCC2* is required for cardiac development by promoting cardiac myocyte differentiation and controlling temporal steps in cardiac differentiation (Hota et al., 2019). More recently, in 2022, Sun et al. found a *de novo SMARCC2* variant in TOF patients, suggesting there may be a link between CHD and deleterious *SMARCC2* variants (Sun et al., 2022). Further research into the pathogenic mechanisms linked with these new variants, and genotype-phenotype associations is required, which may give new therapeutic insights for treating TOF and CHD.

## Discussion

Tetralogy of Fallot is the most common type of cyanotic congenital heart defect and a serious condition that includes four heart abnormalities: a ventricular septal defect, pulmonary stenosis/right ventricular outflow tract obstruction, the aorta overriding the VSD, and right ventricular hypertrophy (Hota et al., 2019). Despite advanced medical interventions, morbidity and mortality for individuals with TOF remain high due to the severe developmental cardiac abnormalities present in this condition. Additionally, the critical causative genes associated with non-syndromic TOF are poorly understood.

Recent progress in identifying the genetic basis for TOF has shown that deleterious variants in *NOTCH1* and *FLT4* play a significant role in TOF and CHD; however, the precise mechanism of these gene variations in TOF remains unknown. In addition, variants in additional cardiac transcription factors were connected to cardiac developmental defects and non-syndromic TOF (Granados-Riveron et al., 2011); Jiang et al., 2013, and (Töpf et al., 2014), suggesting the impacts of these mutants disrupt protein interactions which disrupts the crucial role these proteins play in the evolution of the cardiovascular components.

Recent research findings showed that several rare variants in many different genes might be associated with TOF in individual patients. Additionally, multiple variants in a single individual may act together to perturb cardiac development and cause TOF. Therefore, there may be a large number of genes that cause TOF in only a small number of individuals. These genes may have links to each other in protein interactions or signalling pathways needed for cardiac development. This finding suggests that more research is needed into the pathophysiological mechanisms associated with these new variants and genotype-phenotype associations. This research may be a promising aspect to provide new therapeutic insights for treating TOF and CHD.
